# Breast systemic follicular lymphoma in a man: a case report

**DOI:** 10.1186/1752-1947-6-217

**Published:** 2012-07-23

**Authors:** Elvira La Mantia, Monica Cantile, Giuseppina Liguori, Maurizio Di Bonito, Annarosaria De Chiara, Massimiliano D'Aiuto, Giuseppe Pannone, Renato Franco, Gerardo Botti

**Affiliations:** 1 Pathology Unit, National Cancer Institute, Pascale Foundation, Naples, Italy; 2 Department of Breast Surgery and Oncology, Division of Breast Surgery, National Cancer Institute, Pascale Foundation, Naples, Italy; 3 Department of Surgical Sciences, University of Foggia, Foggia, Italy

**Keywords:** Follicular lymphoma, Male breast, t(14;18) translocation

## Abstract

**Introduction:**

Breast involvement by non-Hodgkin lymphoma is particularly rare in men. We describe the case of a patient with a rapidly growing, painless gynecomastia-like nodule in the left breast. On ultrasonography, the nodule was suspicious for breast carcinoma.

**Case presentation:**

A breast biopsy from a 54-year-old Caucasian man showed the morphoimmunophenotypical features of grade 3 follicular lymphoma. Moreover, fluorescence in situ hybridization analysis showed a t(14,18) translocation suggesting breast involvement by a systemic lymphoma rather than a primary breast lymphoma. The histological diagnosis was subsequently confirmed after nodule excision. Mediastinal and abdominal node involvement was then identified on computed tomography and positron emission tomography scans during staging examinations. Our patient was treated with chemotherapy. After three years our patient experienced a right retro-areolar relapse. He then received two further cycles of chemotherapy but developed a myeloid acute leukemia and, as a result of this, he subsequently died.

**Conclusions:**

The rarity of breast lymphomas, especially in men, and the problems related to the therapeutic choices with these tumors require molecular techniques in association with classical histological diagnosis.

## Introduction

Breast lymphoma is a rare clinicopathological entity that affects both sexes. Approximately one-third of patients with non-Hodgkin lymphoma (NHL) present with extranodal disease [1-3]. Breast involvement could be the expression of a systemic lymphoma or could be the only site of the disease (primary breast lymphoma (PBL)) [3]. A total of 44% of cases of breast lymphoma are primary, while 22% are manifestations of disseminated disease and 29% represent recurrence of pre-existing lymphoma [3]. PBL represents 1% of all non-Hodgkin lymphomas and 0.4% to 0.5% of all breast malignancies [4,5]. Its definition is quite narrow, being relative to patients who had no evidence of disease outside the breast or ipsilateral axillary lymph nodes [6]. Diffuse large B cell lymphoma (DLBCL) is the most common histotype of PBL, while mucosa-associated lymphoid tissue (MALT)-type lymphoma and follicular lymphoma (FL) are less frequent ones. In contrast, secondary breast involvement by lymphoma, although a very rare event, is more frequent for indolent lymphomas, particularly for follicular lymphoma [3]. Systemic FL is usually a disseminated neoplasm, with over two-thirds of cases at stage III or IV at diagnosis [6].

The incidence of breast lymphoma in men is extremely low [7]. In fact fewer than 20 cases have been reported to date [3,8]. Specifically, only two cases of breast FL have been described in men [7,9].

In this report we describe a rare case of breast FL in a man, with a molecular profile typically observed in systemic disease. Staging investigations confirmed systemic disease and relapse after chemotherapy occurred at the contralateral breast. Unfortunately, our patient developed myeloid leukemia, and shortly afterwards died.

## Case presentation

A 54-year-old Caucasian man presented to our facility with a left breast gynecomastia-like nodule. Clinically it was mobile, non-tender and not fixed to the skin or underlying muscles. The results of renal and liver function tests were normal.

Ultrasonography showed a hypoechoic mass with irregular margins, measuring 4.0 × 3.0cm in size. Doppler ultrasound did not show any significant vascularization.

Since a breast carcinoma was suspected, a tru-cut biopsy was performed. From the biopsy results a diagnosis of FL was formulated. The fluorescence in situ hybridization (FISH) demonstration of t(14,18) suggested systemic disease. Two weeks later, the nodule was totally removed, as well as the axillary lymph nodes. The histomorphological features confirmed a diagnosis of lymphoma.

Staging procedures showed positron emission tomography (PET)-positive mediastinal and abdominal lymphoadenomegaly and splenomegaly. A bone-marrow trephine did not show lymphoma infiltration.

Six cycles of R-CHOP (rituximab-cyclophosphamide, hydroxydaunorubicin, Oncovin (vincristine), prednisone/prednisolone) chemotherapy for systemic lymphoma were administered.

Two years later, a new nodule in the right breast was observed. The morphology and immunohistochemistry results of a new biopsy were superimposable to the previous samples. After another six cycles of R-CHOP chemotherapy our patient developed acute myeloid leukemia, from which he died some months later.

All tumor samples from our patient were stained with EE (Ematossilin/Eosin) and then immunohistochemical studies were performed.

The tru-cut biopsy showed proliferation of atypical lymphoid cells with a nodular pattern of growth, with small lymphoid cells (centrocyte type) and centroblast-like cells.

The surgical specimen was represented by a wide excision of the central quadrant extending to the subareolar spaces. Macroscopically the tumor was poorly circumscribed with irregular margins, measuring 4.0 × 3.0cm, with a solid, fleshy cut surface. Histologically the neoplastic proliferation showed similar features to the previous biopsy in in the adipose tissue.

On immunohistochemical analysis the atypical cells were positive for CD20, B cell lymphoma 2 (Bcl2), CD10 and Bcl6 and negative for CD5, CD43 and melanoma associated antigen (mutated) 1 (MUM1) protein; the proliferative index evaluated by Ki67 immunostaining was higher than 50%.

**Figure 1:**
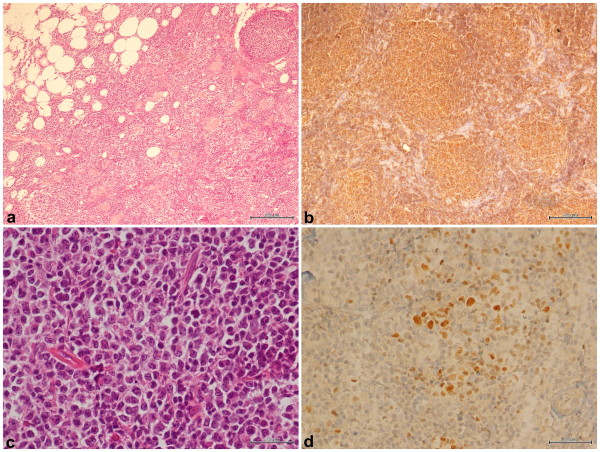
**Immunohistochemistry: follicular lymphoma grade 3, with a diffuse and follicular pattern.** Positive expression of CD10 in tumoral cells. **(a)** EE (Ematossilin/Eosin) (10x); **(b)** CD20 (Magnification 10x); **(c)** EE (Magnification 40x); **(d)** Bcl6 (Magnification 40x).

In the interfollicular areas there were sheets of CD20+/CD10+ atypical lymphoid cells. Centroblasts numbered more than 15. A final diagnosis of grade 3 FL was made (Figure 1).

Histology results of the right breast relapse sample showed similar features to previous breast samples.

FISH analysis for the t(14;18)(q32;q21) translocation was performed on the tru-cut biopsy of the primary breast nodule using the Vysis LSI IGHSpectrumGreen/LSI BCL2 SpectrumOrange probe set (Vysis, Downers Grove, IL, USA). This probe set uses the dual-color, dual-fusion strategy and consists of a mixture of locus-specific fluorophore-labeled DNA probes containing sequences homologous to the *IgH* and *Bcl2* genes.

**Figure 2:**
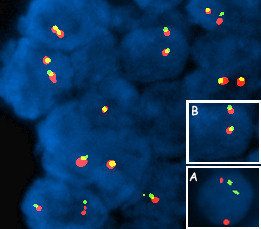
**Representative fluorescence in situ hybridization (FISH) patterns with LSI IGH/BCL2 DualColor, Dual Fusion Translocation Probe (Vysis).** This probe set is a mixture of locus-specific immunoglobulin heavy chain (IgH) probe labeled with SpectrumGreen (Vysis) and a locus-specific probe for B cell leukemia/lymphoma 2 (Bcl2) labeled with SpectrumOrange (Vysis). 1R1G2F classic dual-fusion (D-FISH) pattern in a balanced t(14;18)(q32;q21)-IgH/Bcl2+ case. (A) Translocated signal; (B) non-translocated signal.

FISH analysis again showed the presence of t(14;18) translocation on the surgical sample from the left breast and the biopsy of the right relapse (Figure 2).

Then, after DNA extraction from a paraffin embedded sample, we performed multiplex polymerase chain reaction (PCR) for the identification of the rearrangement of heavy chain (IgH) CDR1-CDR3.

**Figure 3:**
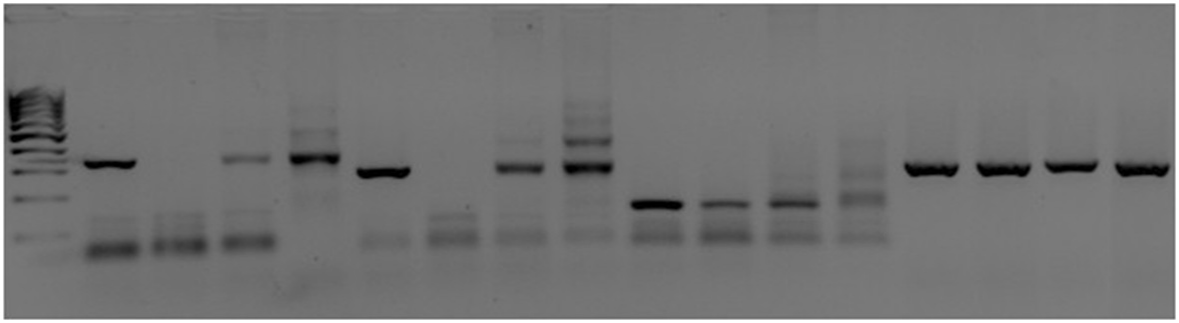
**Representative gel photo of polymerase chain reaction (PCR) for immunoglobulin heavy chain rearrangements.** Lane 1 represents the 100-bp DNA ladder (Invitrogen, Carlsbad, CA, USA); lane 2 represents FR1 rearrangement on monoclonal control; lane 3 represents FR1 rearrangement on internal sample; lane 4 represents FR1 rearrangement on follicular lymphoma sample; lane 5 represents FR1 rearrangement on polyclonal control; lanes 6 to 9 represent FR2 rearrangement on the same set of samples; monoclonal control; lanes 10 to 13 represent FR3 rearrangement on the same set of samples. The last four lanes represent β globin gene amplification.

DNA purified from the paraffin sample was of good quality as shown by the presence of discrete bands for both FR1, FR2 and FR3 rearrangements, indicative of a B monoclonal pattern (Figure 3).

## Discussion

Breast lymphoma is a rare entity. Primary breast lymphomas represent 44% of cases and secondary involvement by systemic disease accounts for more than 50% of cases [3]. PBLs account for 1.7% to 2.2% of all extranodal lymphomas and 0.38% to 0.7% of all NHL [10-13]. The most described histotype is DLBCL [1]. The age range of incidence is between nine and 85 years. Wiseman and Liao [6] reported three criteria for diagnosis of primary NHL of the breast. Adequate pathological evaluation, presence of both mammary tissue and lymphoma infiltrate in close association, and exclusion of either systemic lymphoma or previous extramammary lymphoma. Breast involvement by systemic lymphoma is quite rare. It could occur either at initial diagnosis or relapse [3]. Secondary lymphomas in the breast are typical indolent lymphomas, particularly follicular lymphomas. Both PBL and secondary breast lymphomas are rarely described in men [7-9]. In fact, less than 20 cases have been described [8]. The recognition of breast lymphoma in men is very difficult because of the extensive involution of the male breast gland. In our patient’s case the lesion first involved the retro-areolar left parenchyma and then the retro-areolar right tissue, demonstrating a specific tropism. To the best of our knowledge only two cases of breast FL in men have been described. In particular our patient’s case represents presentation of systemic lymphoma as a gynecomastic-like nodule. Moreover the presence of t(14;18) gave the suggestion of systemic disease rather than a PBL, as confirmed by staging procedures in our patient. In fact, incidence of t(14;18) frequently presents in systemic disease [6].

## Conclusions

In summary, follicular lymphoma of the breast in a man is an uncommon histologic subtype. The presence of a t(14;18) translocation in our patient’s case also suggests a disseminated disease rather than a primary lymphoma, as has already been described in other organs.

## Consent

Written informed consent was obtained from the patient’s next of kin for publication of this manuscript and any accompanying images. A copy of the written consent is available for review by the Editor-in-Chief of this journal.

## Competing interests

The authors declare that they have no competing interests.

## Authors’ contributions

GB and RF were responsible for interpretation of the case report. ELM, MDB, and MD were responsible for provision of study materials or details regarding our patient. MC, GL, ADC, GL and RF collected and assembled data and samples for FISH and immunohistochemical analysis. MC was responsible for PCR clonality analysis. EML, MDB and ADC were responsible for immunohistochemical evaluation. GL and RF were responsible for FISH evaluation. All authors were involved in manuscript writing and provided final approval for the manuscript.
